# Supplementary material for: LINE-1 regulates cortical development by acting as long non-coding RNAs
Source: Nat Commun. 2023 Aug 17;14:4974. doi: 10.1038/s41467-023-40743-7 (PMC10435495; doi:10.1038/s41467-023-40743-7)
Supplement: Supplementary file 1 — Supplementary Information [file 41467_2023_40743_MOESM1_ESM.pdf]

Supplementary Information

LINE-1 regulates cortical development by acting as long non-coding RNAs

Damiano Mangoni[1], Alessandro Simi[1], Pierre Lau[1], Alexandros Armaos[1], Federico Ansaloni[1], Azzurra Codino[1], Devid Damiani[1], Lavinia Floreani[2], Valerio Di Carlo[1], Diego Vozzi[1], Francesca Persichetti[3], Claudio Santoro[3], Luca Pandolfini[1], Gian Gaetano Tartaglia[1], Remo Sanges[1,2]*, and Stefano Gustincich[1]*

*Corresponding authors

[1] Central RNA Laboratory, Istituto Italiano di Tecnologia (IIT), Genova, Italy
[2] Area of Neuroscience, International School for Advanced Studies (SISSA), Trieste, Italy
[3] Department of Health Sciences and Research Center on Autoimmune and Allergic Diseases (CAAD), University of Piemonte Orientale (UPO), Novara, Italy

Address correspondence and requests to:

Stefano Gustincich, Ph.D.,
Senior Researcher – Principal Investigator,
Deputy Director for Technologies for Life Science (LifeTech) –
Director Central RNA Lab
Center for Human Technologies (CHT),
RNA central Lab. IIT Central Research Labs Genova.
Via Enrico Melen 83, 16152 Genova, Italy.
Office phone: +39 010 2897620
E-mail address: Stefano.Gustincich@iit.it

Remo Sanges, Ph.D.,
Associate Professor in Bioinformatics and Genomic Data Analysis
Area of Neuroscience,
International School for Advanced Studies (SISSA), 34136, Trieste, Italy
E-mail address: remo.sanges@gmail.com

The authors have declared that no conflict of interest exists.

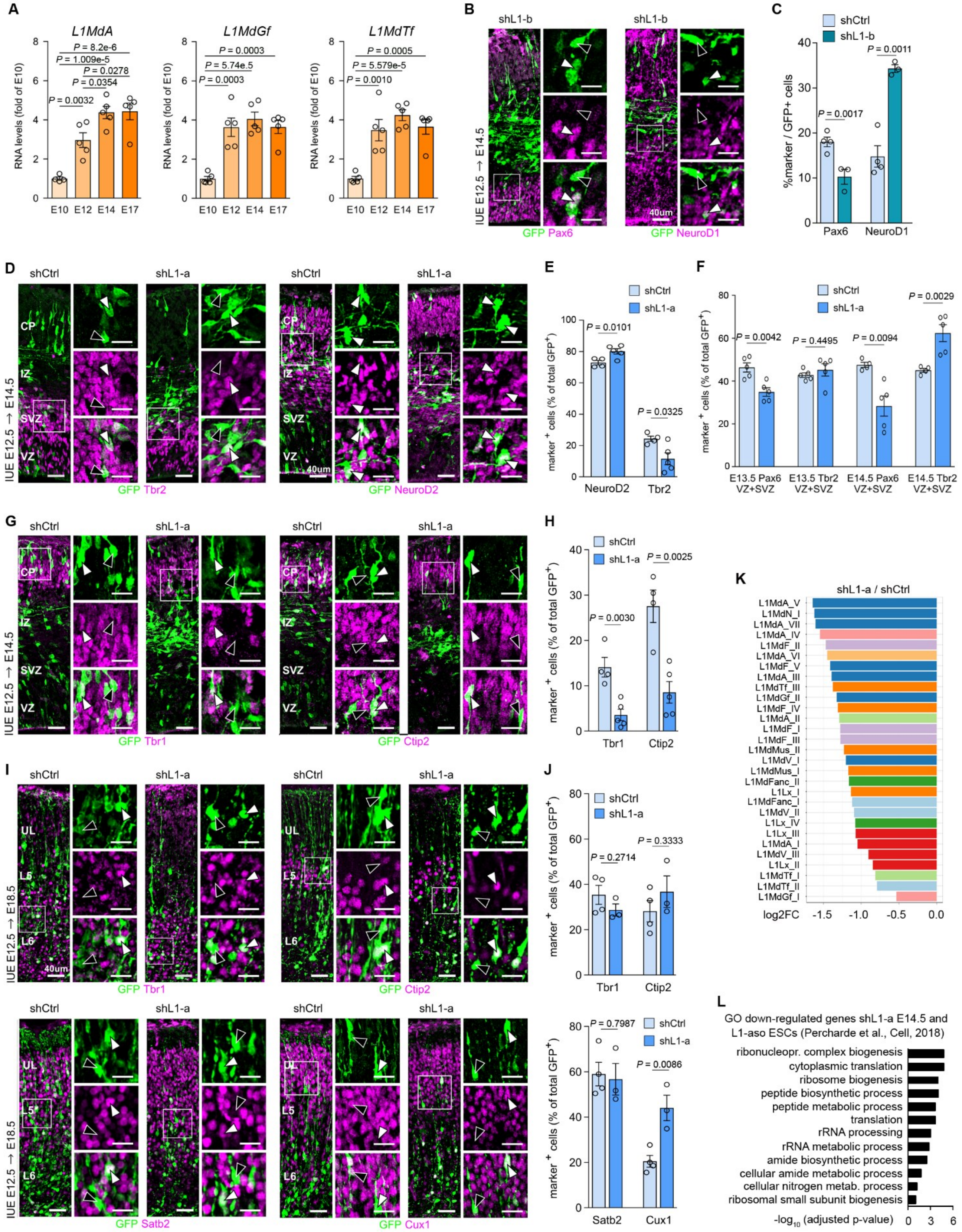

Supplementary Figure S1. L1 silencing alters the development of the mouse brain cortex. a, Expression levels of L1 subfamilies in the developing mouse brain cortex. Data are mean ± s.e.m.; *n* = 5 independent biological samples. One-way ANOVA with Tukey's multiple comparisons test. **b**, Immunofluorescence staining for neuronal progenitor (Pax6) and pro-neuronal (NeuroD1) markers

at E14.5 after i.u.e. of shL1-b. **c**, Quantification of GFP$^+$/marker$^+$ cells at E14.5 after i.u.e. with shL1-b. Data are mean ± s.e.m.; *n* = 4 shCtrl, *n* = 3 shL1-b. Two-sided unpaired t-test. **d**, **g**, **i**, Immunofluorescence staining for Tbr2 and NeuroD2 at E14.5 (**d**), Tbr1 and Ctip2 at E14.5 (**g**), and Tbr1, Ctip2, Satb2 and Cux1 (**i**) at E18.5. **e**, **f**, **h**, **j**, Quantification of GFP+/marker$^+$ cells for NeuroD2 and Tbr2 at E14.5 (**e**), site-specific quantification of double positive (GFP$^+$/marker$^+$) cells at E13.5 and E14.5 for Pax6 and Tbr2 (**f**), Tbr1 and Ctip2 at E14.5 (**h**), Tbr1, Ctip2, Satb2 and Cux1 at E18.5 (**j**). Data are mean ± s.e.m.; E13.5: *n* = 5 each; E14.5: *n* = 4 shCtrl, *n* = 5 shL1-a. SZ, subventricular zone; VZ, ventricular zone; IZ, Intermediate zone; CP, cortical plate. Two-sided unpaired t-test. **k**, log2FC of L1 subfamilies transcript levels from RNA-Seq experiment in E14.5 cortex i.u.e. with shL1-a compared to shCtrl, analyzed by TEspeX. **l**, Top GO terms under the biological process category for down-regulated genes by L1-targeting antisense oligonucleotide (L1-aso) in mouse embryonic stem cells (mESC). *p* values were determined by gprofiler2 using a default hypergeometric test and correction for multiple testing has been performed by the g:SCS algorithm. Source data are provided as a Source Data file.

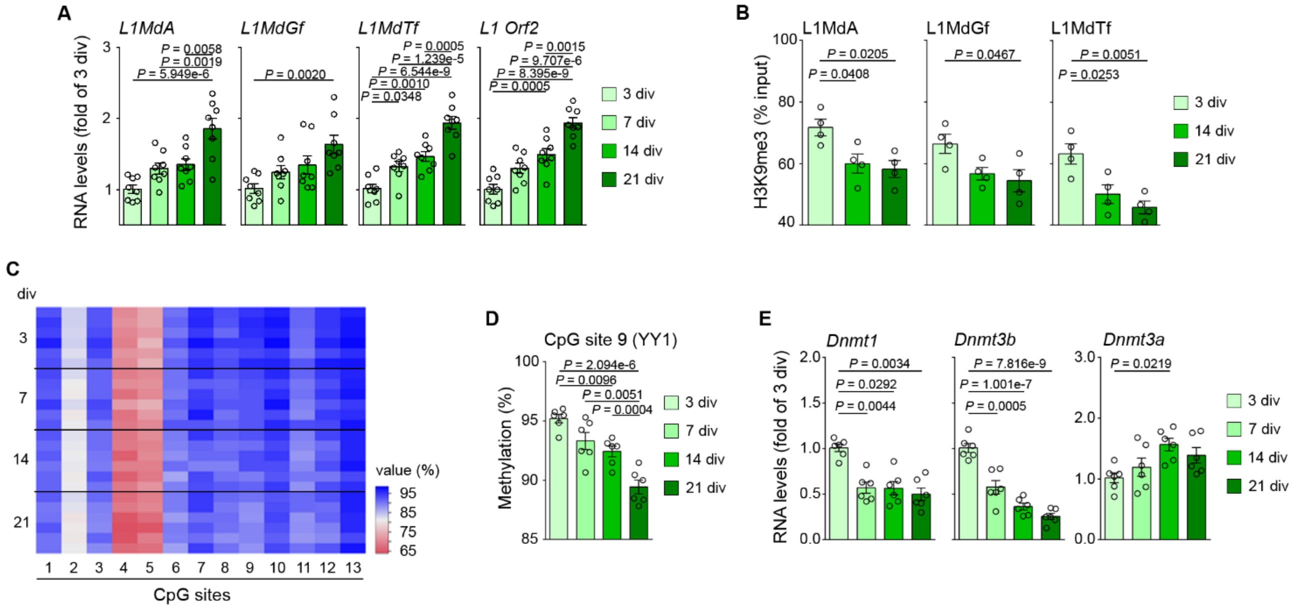

Supplementary Figure S2. L1 expression is epigenetically regulated in developing mouse cortical neurons. a, Expression levels of L1 families (L1MdA, L1MdGf and L1MdTf) and conserved regions (L1 Orf2) in 3, 7, 14 and 21 div cultured cortical neurons. RNA fold enrichment is normalized on the early (3 div) time point. *n* = 8 independent biological samples. **b**, Chromatin Immunoprecipitation (ChIP) of H3K9me3 and enrichment for L1 families promoter in 3, 14 and 21 div cortical neurons. Fold enrichment is expressed as percentage of the input. *n* = 3 independent biological samples. **c**, Heatmap of the percentage methylation levels of L1MdTf promoter in 3, 7, 14 and 21 div cortical neurons. **d**, Percentage methylation levels of the CpG site 9 containing the YY1 binding site in 3, 7, 14 and 21 div cortical neurons. *n* = 6 independent biological samples. **e**, Expression levels of *Dnmt1*, *Dnmt3b* and *Dnmt3a* in 3, 7, 14 and 21 div cortical neurons. RNA fold enrichment is normalized on the early (3 div) time point. *n* = 6 independent biological samples. For **a**, **b**, **d** and **e**, data are mean ± s.e.m. One-way ANOVA with Tukey's multiple comparison test. Source data are provided as a Source Data file.

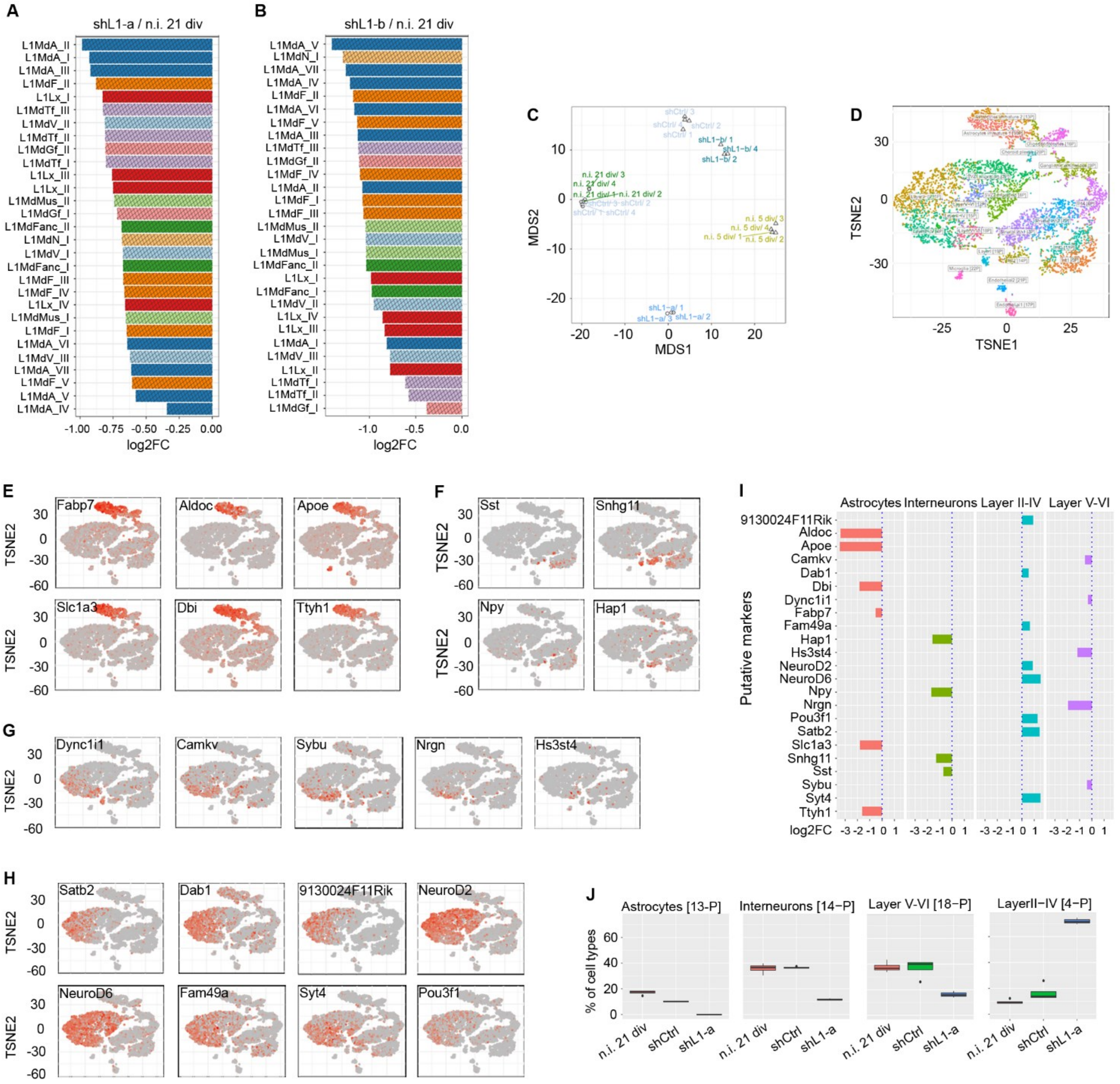

Supplementary Figure S3. Prediction of altered cell type composition by L1 silencing in cortical cultures. a, log2FC of L1 subfamilies transcript levels from RNA-Seq experiments in cultured cortical cells infected with shL1-a (**a**) or shL1-b (**b**) compared to n.i. samples, analyzed by TEspeX. **c**, Multidimensional scaling (MDS) plot of RNA-Seq experiments on cultured cortical cells non-infected (n.i.) or infected with shCtrl, shL1-a or shL1-b. The different biological groups are shown in different colors. The shape of the points indicates the sequencing run (circle = run 1, triangle = run 2). **d**, Single-cell Drop-seq from P0 brain cells showing brain-area specific gene expression. **e, f**, **g**, **h**, TSNE plots of mouse brain cells of putative gene markers for astrocytes (*Fabp7*, *Slc1a3*, *Dbi*, *Aldoc*, *Apoe* and *Tthy1*) **e**), interneurons (*Sst*, *Npy*, *Snhg11* and *Hap1*) **f**), layer V-VI cells (*Dync1i1*, *Camkv*, *Sybu*, *Nrgn* and *Hs3st4*) **g**) and layer II-IV cells (*Satb2*, *Dab1*, *9130024F11Rik*, *NeuroD2*, *NeuroD6*, *Fam49a*, *Syt4* and *Pou3f*) **h**) inferred by MuSiC deconvolution method as down-regulated or up-regulated in n.i. 21 div cortical neurons, or infected with shCtrl or shL1-a. Grey color indicates a low expression and red a higher expression of the gene marker. **i**, log2 fold changes of putative markers for four cell clusters (astrocytes, interneurons, layer V-VI and layer II-IV) responding to

shL1-a treatment compared to control cells. **j**, Percentage proportions of four cell clusters (astrocytes, interneurons, layer V-VI and layer II-IV) detected by MuSiC deconvolution method with significant proportion changes in shL1-a compared to control cells. $n = 4$ independent biological replicates for n.i. 21 div and shCtrl groups; $n = 3$ independent biological replicates for shL1-a group. The boxes show the interquartile range (IQR), the central line represents the median, the whiskers add 1.5 times the IQR to the 75 percentile (box upper limit) and subtract 1.5 times the IQR from the 25 percentile (box lower limit). One-way ANOVA test. Source data are provided as a Source Data file.

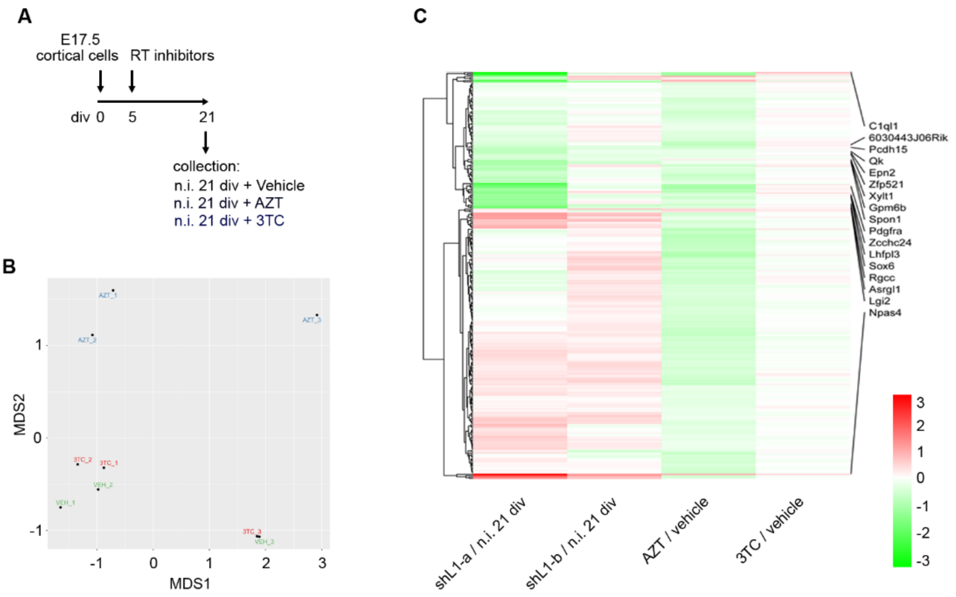

Supplementary Figure S4. Effect of the pharmacological inhibition of L1 reverse transcriptase in mouse cortical neurons. a, Schematic of the timeline of cortical cells treatment with reverse transcriptase inhibitors. **b**, Multidimensional scaling (MDS) plot of RNA-Seq experiments on cultured cortical cells treated with vehicle, AZT, or 3TC. The different biological groups are shown in different colors. **c**, Heatmap of the log2 fold changes of DEGs in cells treated with AZT or 3TC compared to vehicle, and to cells infected with shL1-a and shL1-b compared to n.i. The rows correspond to the significant genes and the columns to the group comparisons.

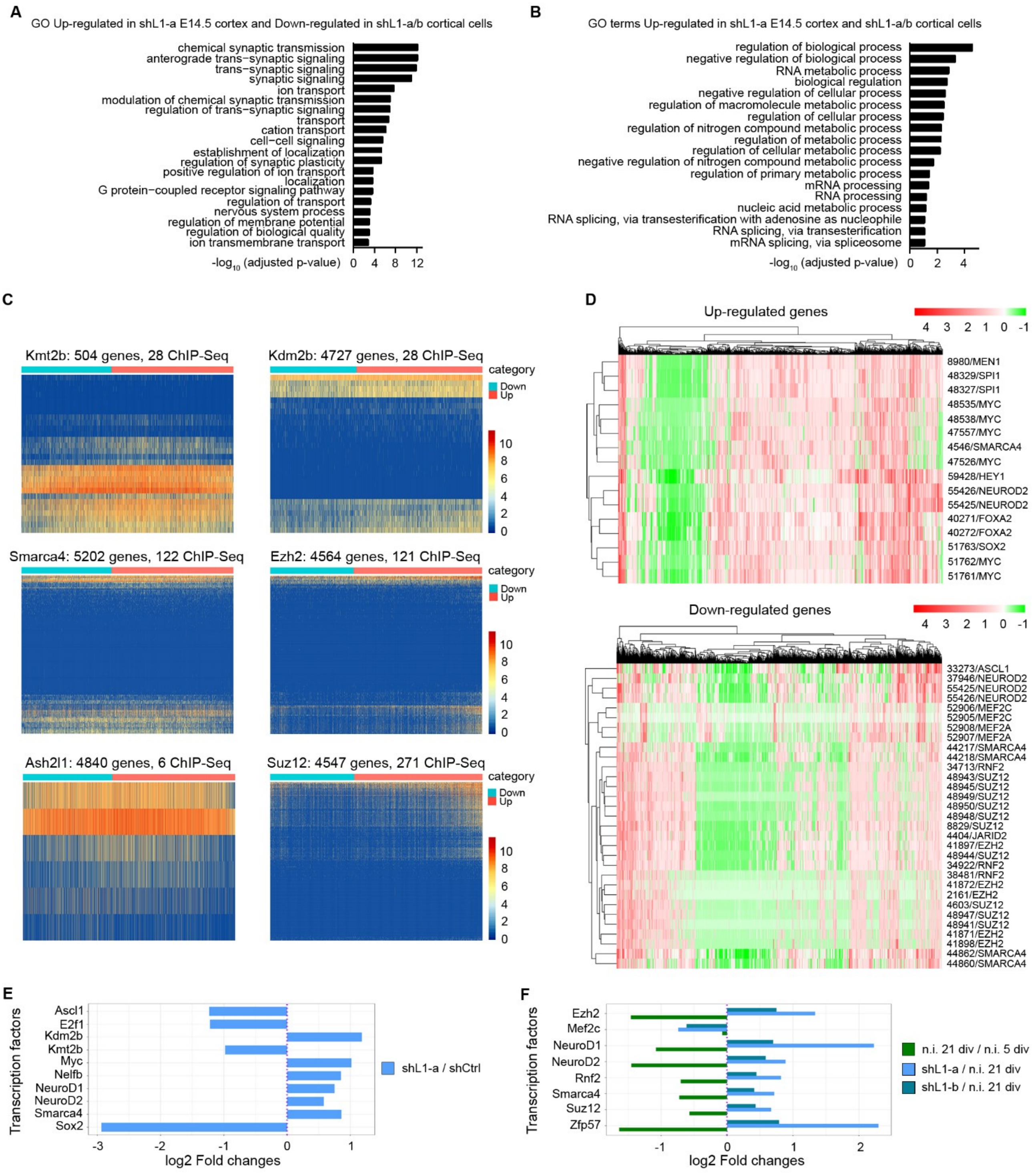

Supplementary Figure S5. Prediction of transcriptional regulators of differentially expressed genes by shL1 in vivo and in vitro. a, b, GO analysis under the biological process category of genes up-regulated in shL1-a E14.5 i.u.e cortex and down-regulated in shL1-s treated cortical cells (**a**) and of up-regulated genes in both shL1-a E14.5 i.u.e. cortex and shL1-s infected cortical cells datasets (**b**). **c,** Heatmaps representing the log2 MACS2 binding scores from publicly available ChIP-Seq experiments (rows) for each gene (columns) ordered by log2 fold changes obtained after shL1-a treatment of E14.5 mouse cortex. The horizontal ribbons categorize the up- and down-regulated genes. The most down-regulated genes are on the left side of the heatmap and the most up-regulated

ones are on the right side. The total number of differentially expressed genes is 6185 (RankComp V2 test, FDR <= 0.05). Genes with ChIP-Seq peaks located within a maximum distance of 1 kb from the annotated TSS are represented in the heatmaps. **d**, In the heatmaps, each column corresponds to a differentially expressed gene in shL1-a and shL1-b treated cultured cortical cells, and each row to a Cistrome DB ChIP-Seq experiment as indicated by the Cistrome DB ID and the name of the transcriptional regulator (right side). A red color corresponds to a high regulatory score. A green color is used for a low regulatory score. The upper panel corresponds to the up-regulated gene set and the lower panel to the down-regulated gene set respectively. For **c** and **d**, regulatory scores were calculated by Lisa v2.2.4 software. **e**, **f**, log2 fold changes of differentially expressed transcriptional regulators (FDR <= 0.05, limma test) obtained from RNA-Seq experiments for shL1-a versus shCtrl in the E14.5 i.u.e. (**e**), or for shL1-a and shL1-b conditions after comparing to the 21 div n.i. in culture cortical cells (**f**). A positive log2 fold change indicates an up-regulation by the shRNA whereas a negative log2 fold change represents a down-regulation of the inferred transcriptional regulator.

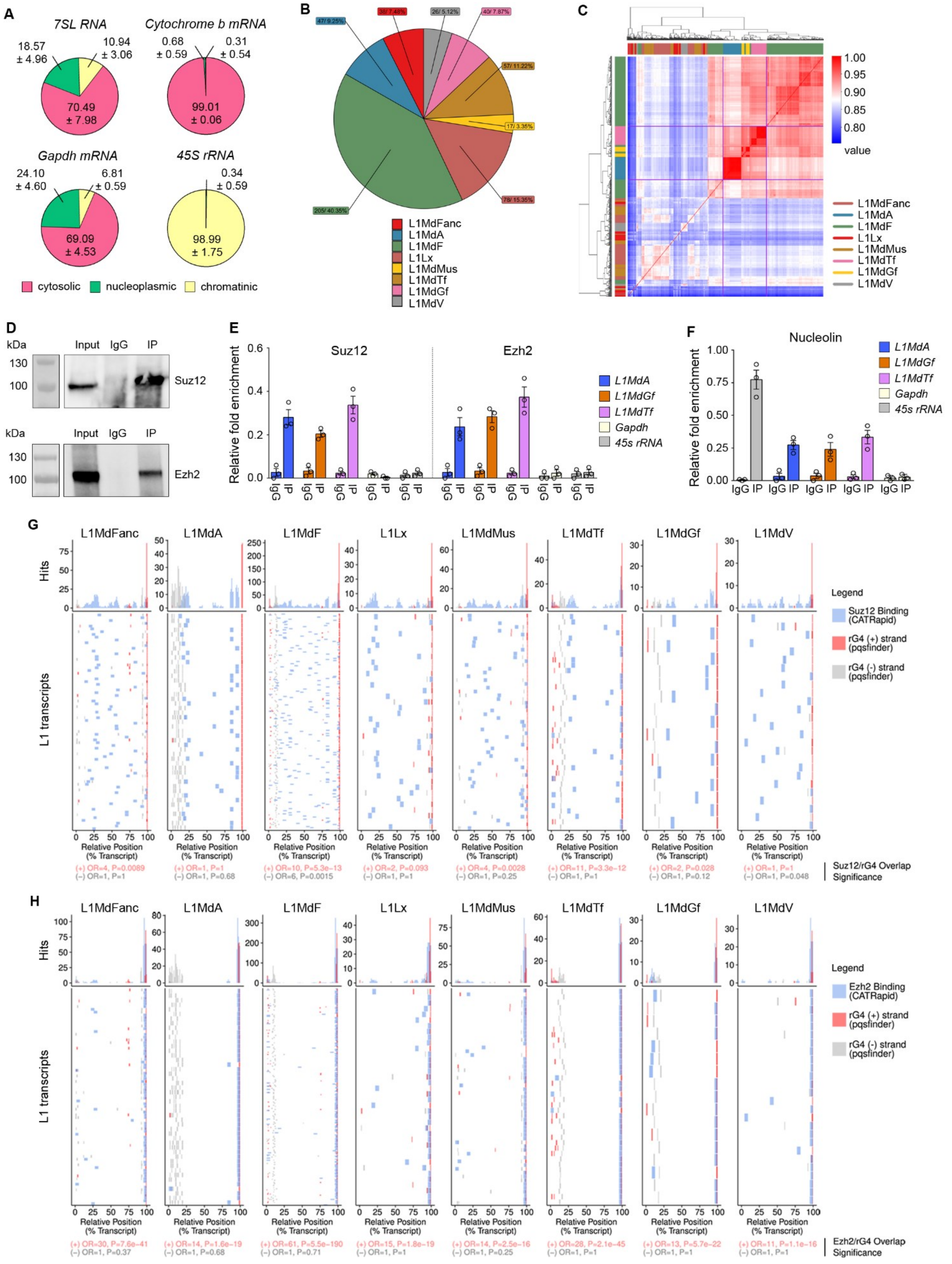

Supplementary Figure S6. Putative Suz12 and Ezh2 binding sites on L1 RNAs overlap with predicted G-quadruplex sequences. a, Cytosolic, nucleoplasmic and chromatinic abundance of *7sL*,

Gapdh, *Cytochrome b* and *45s* RNAs in 21 div cells. RNA levels are expressed as percentage of total RNA. *n* = 3 independent biological samples. Data are mean ± s.e.m. **b**, Pie chart showing the percentage proportion of the L1 sequences per evolutionary time-point used for *cat*RAPID analysis. **c**, Heatmap showing the alignment among all the L1 sequences clustered on the basis of the evolutionary time-points submitted to catRAPID analysis. Higher alignment scores are in red and lower in blue. **d**, **e**, RNA immunoprecipitation (IP) for Suz12 and Ezh2 in total RNA fraction of 21 div cells. Western blot showing protein enrichment after IP (**d**). Fold enrichment of L1 transcripts and negative controls (*Gapdh* and *45s rRNA*) in Suz12 and Ezh2 IP (**e**). **f**, RNA IP for nucleolin and fold enrichment of *45s rRNA*, L1 subfamilies transcripts and *Gapdh* in nucleolin IP. For **e** and **f**, RNA levels are relative to input control. *n* = 3 independent biological samples. Data are mean ± s.e.m. **g**, **h**, Distribution of the putative Suz12 (**g**) and Ezh2 (**h**) binding sites as predicted by cat*RAPID* (blue) along with the putative G-quadruplex (rG4) sequences detected by pqsfinder on the plus (red) or minus (gray) strand of L1 transcripts, grouped by their evolutionary time-point. The top graph diplays the cumulative distribution of these features on a L1 meta-transcript. Odds Ratios (OR) and P values show the degree of statistical significance of the overlap between cat*RAPID*-predicted binding sites and either sense (+; red) or antisense (-; gray) rG4 motifs. The statistical significance of the association between predicted rG4 sequences and protein binding sites was assessed using the mergePeaks function within HOMER suite. Source data are provided as a Source Data file.
