# Supplementary material for: LINE-1 regulates cortical development by acting as long non-coding RNAs
Source: Nat Commun. 2023 Aug 17;14:4974. doi: 10.1038/s41467-023-40743-7 (PMC10435495; doi:10.1038/s41467-023-40743-7)
Supplement: Supplementary file 3 — Description of Additional Supplementary Files [file 41467_2023_40743_MOESM3_ESM.pdf]

Description of Additional Supplementary Files

Supplementary Data 1: List of oligos and antibodies

Supplementary Data 2: DEGs and GOs shL1-a at E14 and overlap with mESCs

Supplementary Data 3: DEGs and GOs shL1-a/b in 21 div cells with H3K27me3 mark ENCODE

Supplementary Data 4: Off-targets of shL1-a/b among down-regulated genes

Supplementary Data 5: DEGs AZT treatment in 21 div cells

Supplementary Data 6: GOs overlaps shL1-a/b at E14 and 21 div cells

Supplementary Data 7: ChIP-Seq Diffbind and GOs for H3K27me3 and Ezh2

Supplementary Data 8: L1 sequences for catRAPID omicsV2 0210225 annotation

Supplementary Data 9: Ranking catRAPID omicsV2 interaction propensity 1946 RBPs to L1 RNA sequences
