# Supplementary material for: LINE-1 regulates cortical development by acting as long non-coding RNAs
Source: Nat Commun. 2023 Aug 17;14:4974. doi: 10.1038/s41467-023-40743-7 (PMC10435495; doi:10.1038/s41467-023-40743-7)
Supplement: Supplementary file 13 — Reporting Summary [file 41467_2023_40743_MOESM13_ESM.pdf]

nature portfolio

Corresponding author(s): Stefano Gustincich, Remo Sanges

Last updated by author(s): Jul 7, 2023

Reporting Summary

Nature Portfolio wishes to improve the reproducibility of the work that we publish. This form provides structure for consistency and transparency in reporting. For further information on Nature Portfolio policies, see our Editorial Policies and the Editorial Policy Checklist.

Statistics

For all statistical analyses, confirm that the following items are present in the figure legend, table legend, main text, or Methods section.

n/a	Confirmed	
[ ]	[x]	The exact sample size (*n*) for each experimental group/condition, given as a discrete number and unit of measurement
[ ]	[x]	A statement on whether measurements were taken from distinct samples or whether the same sample was measured repeatedly
[ ]	[x]	The statistical test(s) used AND whether they are one- or two-sided<br>*Only common tests should be described solely by name; describe more complex techniques in the Methods section.*
[x]	[ ]	A description of all covariates tested
[ ]	[x]	A description of any assumptions or corrections, such as tests of normality and adjustment for multiple comparisons
[ ]	[x]	A full description of the statistical parameters including central tendency (e.g. means) or other basic estimates (e.g. regression coefficient) AND variation (e.g. standard deviation) or associated estimates of uncertainty (e.g. confidence intervals)
[ ]	[x]	For null hypothesis testing, the test statistic (e.g. *F*, *t*, *r*) with confidence intervals, effect sizes, degrees of freedom and *P* value noted<br>*Give P values as exact values whenever suitable.*
[x]	[ ]	For Bayesian analysis, information on the choice of priors and Markov chain Monte Carlo settings
[x]	[ ]	For hierarchical and complex designs, identification of the appropriate level for tests and full reporting of outcomes
[x]	[ ]	Estimates of effect sizes (e.g. Cohen's *d*, Pearson's *r*), indicating how they were calculated

Our web collection on statistics for biologists contains articles on many of the points above.

Software and code

Policy information about availability of computer code

Data collection	DRAGEN Germline Pipeline v3, Nikon software version 4.11.0, CFX Maestro Software 2.3, Sequencing Analysis Software for Windows 10, iBright Analysis Software
Data analysis	For RNA-seq analysis on cultured cortical neurons: sequences were aligned onto the mm10 mouse build using STAR aligner (version 2.7.3a), Gene expression was quantified with featureCounts in stranded mode (Subread version 2.0.0) and the mouse Gencode GTF annotation (version M25), distances between samples were calculated based on regularized log transformed counts (rlog function in DESeq2 version 1.30.1), distances were transformed into a dissimilarity matrix by classical Multidimensional Scaling (MDS, cmdscale in R version 4.0.5), differential gene expression (DGE) analysis was made with limma (version 3.46.0).<br>For RNA-seq on FAC-sorted cells from in utero electroporated cortexes: reads were counted with featureCounts in unstranded mode and DGE analysis was made using the RankComp V2 algorithm (REOA version 0.1), heatmaps were rendered using pheatmap (version 1.0.12) and annotated with ComplexHeatmap (version 2.6.2).<br>TE expression was measured by using TEspeX (--strand reverse, https://github.com/fansalon/TEspeX/).<br>Gene ontology (GO) enrichment analyses have been performed using gprofiler2 (version 0.2.1), correction for multiple testing has been performed by using the g:SCS algorithm. The inference of transcriptional regulators for the in vitro and in utero experiments was made with Lisa (version 2.2.4 under Python 3.8). Additional evidence for Lisa inferred transcriptional regulators was obtained from ChIP-Seq data in the ChIP-Atlas database (https://chip-atlas.org), using the Enrichment Analysis module to query mouse experiments with MACS2 ChIP-Seq peaks.<br>The quantification of reads mapping to different L1 subfamilies in the shL1-a and shL1-b in-vitro experiment was done using TEspeX (version 1.0.3).<br>For single-cell Drop-seq data from P0 (birth) C57BL/6J brain cortices quality control (QC) metrics were done using scater (version 1.14.6) and single cells with a high mitochondrial content, or low library size or low number of expressed genes, were removed from the single-cell analysis. The genes with less than 0.005 counts on average among the 7111 single cells were filtered-out and normalization was done with

scran (version 1.14.6).
The percentage of cell types in the in-vitro model was estimated by MuSiC (version 0.1.1) and further analyzed for putative cell markers using Signac (version 0.0.9) and Seurat (version 4.0.2).
For protein-RNA interactions we used the http://s.tartaglialab.com/page/catrapid_group and http://s.tartaglialab.com/page/catrapid_omics2_group to identify interactions regions and recurrent motifs.
rG4 sequences on L-1 transcripts were predicted using pqsfinder.
To identify potential off-target transcripts recognised by the shL1a and shL1b assays, the nucleotide sequences of the two assays were aligned to the mm10 reference transcriptome (gencode vM25) by using bowtie (v1.2.3) allowing 0 mismatches and selecting only end-to-end matches, as of bowtie default (parameters: -f -S -y -a -v 0).
ChIP-Seq reads were aligned to the mm10 mouse genome using the Bowtie2 aligner. The unmapped reads, not primary aligned reads, and reads aligned with a MAPQ quality score below 30 were filtered out with samtools. The duplicated reads were marked with Picard, then removed and the reads mapped as proper pairs were sorted into a BAM file. The BAM files were transformed into bigWig with deepTools for visualization purposes in the UCSC genome browser and into BEDPE format with bedtools. The metagene analysis was performed using SeqCode, to directly visualize the distribution of the aligned reads present in the BAM files onto gene models. The metagenes were defined by the mm10 RefGene database and the plot was made after counting the number of reads along the region of each gene and averaging this number by the number of genes and the total number of mapped reads. A flanking region of 3 kbs upstream and downstream of the gene body was also considered during the counting. The peak calling was done with epic2, a re-implementation of SICER, on the BEDPE files using matched input samples as control and a FDR threshold of 0.01 when calling the peaks or islands. The differentially bound sites obtained during the epic2 calls were found using DiffBind and a FDR of 0.05. The data was normalized by default, based on sequencing depth and the differential analysis was also performed by default, using DESeq2 and a FDR threshold of 0.05.
The statistical significance of the association between predicted rG4 sequences and protein binding sites was assessed using the mergePeaks function within HOMER suite (Hypergeometric Optimization of Motif EnRichment).
Flurescence images were analyzed with Nikon software version 4.11.0 and ImageJ version 1.53i.
GraphPad Prism 7 was used for t-test and ANOVA with multiple comparisons tests.
For RNA-Seq experiments and ChIP-Seq, the statistical treatments were done with R version 4.0.3 and Bioconductor release 3.12, or R version 4.2 and Bioconductor release 3.16, respectively

For manuscripts utilizing custom algorithms or software that are central to the research but not yet described in published literature, software must be made available to editors and reviewers. We strongly encourage code deposition in a community repository (e.g. GitHub). See the Nature Portfolio guidelines for submitting code & software for further information.

Data

Policy information about availability of data

All manuscripts must include a data availability statement. This statement should provide the following information, where applicable:

- Accession codes, unique identifiers, or web links for publicly available datasets
- A description of any restrictions on data availability
- For clinical datasets or third party data, please ensure that the statement adheres to our policy

All data and materials supporting the findings of this study are available in the main text or the Supplementary Information, and from the corresponding authors upon request. The raw RNA-Seq and ChIP-seq data have been deposited at ENA (European Nucleotide Archive) under the series accession codes PRJEB48280, PRJEB48281 and PRJEB58556.
Sequence alignment was done using the mouse reference genome GRCm38 [https://www.ncbi.nlm.nih.gov/assembly/GCF_000001635.20/].
Inference of transcriptional regulators was done using public ChIP-Seq data form Cistrome DB (http://cistrome.org/db/#/) and ChIP-Atlas database (https://chip-atlas.org).
Source data underlying Figs. 1b,1d,1f,1h; 2c,2g,2i; 3c,3d; 4d,4e; S1A,S1C,S1E,S1F,S1H,S1J; and Supplementary Figs. S2A,S2B,S2C,S2D,S2E; S3J; S6A,S6D,S6E,S6F are provided with this paper as a Source Data file.

Research involving human participants, their data, or biological material

Policy information about studies with human participants or human data. See also policy information about sex, gender (identity/presentation), and sexual orientation and race, ethnicity and racism.

Reporting on sex and gender	Not applicable
Reporting on race, ethnicity, or other socially relevant groupings	Not applicable
Population characteristics	Not applicable
Recruitment	Not applicable
Ethics oversight	Not applicable

Note that full information on the approval of the study protocol must also be provided in the manuscript.

Field-specific reporting

Please select the one below that is the best fit for your research. If you are not sure, read the appropriate sections before making your selection.

[x] Life sciences [ ] Behavioural & social sciences [ ] Ecological, evolutionary & environmental sciences

Life sciences study design

All studies must disclose on these points even when the disclosure is negative.

Sample size	Sample size calculation was not performed. Sample size for each experimental protocol was chosen based on extensive experience of working with in utero electroporation, primary cortical neurons, RNA-seq experiments, ChIP-seq experiments, as well as animal availability. For in utero electroporation experiments, sample size choice is based on standards in the field (dal Maschio M. et al. Nat Commun 3, 960 (2012). https://doi.org/10.1038/ncomms1961 and Szczurkowska, J. Nat Protoc 11, 399–412 (2016). https://doi.org/10.1038/nprot.2016.014).<br>A number of independent biological replicates ranging from 3 to 8 was used for in vivo and in vitro experiments, depending on the type of experiment. Our statistical analysis indeed showed significant differences in the experimental and control conditions.
Data exclusions	In this study all data obtained from experiments are presented, also in the case of negative results.
Replication	The experimental protocols performed on primary isolated cortical neurons were performed in triplicate for each biological sample (n = 3-8, depending on the experimental protocol).<br>Experiments performed in vivo on electroporated brain cortexes were performed with one technical replicate for each biological replicates (n = 3-6, depending on experimental groups and developmental stages).<br>RNA-seq experiments on primary isolated cortical neurons were performed with one technical replicate for each biological replicate (n = 4).<br>RNA-seq experiments on FAC-sorted cells from in utero electroporated cortexes were performed with 4 technical replicates for each batch of cells collected after sorting (n = 3 batches of 200 cells, from 1 pool of 2 embryos).<br>RNA-seq experiments on cortical cells tretaed with L1-RT inhibitors were performed with one technical replicate for each biological replicate (n = 3).<br>ChIP-seq experiments on cortical cells were perfomed with one technical replicate for each biological replicate (n = 2).<br>For each experimental protocol, all attempts at verification were successful.
Randomization	The experimental procedures on in vitro cultured mouse cells do not present any obvious need for randomization since cells treated with control or test iRNAs belong to the same source; each independent biological sample is a cell population deriving from the brain of a pool of different embryos belonging to same litter (same mother).<br>For in vivo experiment of in utero electroporation, pregnant female mice were randomly allocated to be treated with Ctrl or test iRNAs.
Blinding	Experiments and data analysis performed in this study (in vitro experiments, in vivo experiments, library preparation for RNA-seq and ChIP-seq, gene expression analysis, TE expression analysis and catRAPID predictions) were performed by separate researchers to minimize bias. Experiments performed by a single researcher were performed without blinding.

Reporting for specific materials, systems and methods

We require information from authors about some types of materials, experimental systems and methods used in many studies. Here, indicate whether each material, system or method listed is relevant to your study. If you are not sure if a list item applies to your research, read the appropriate section before selecting a response.

Materials & experimental systems

n/a	Involved in the study	
☐	☒	Antibodies
☒	☐	Eukaryotic cell lines
☒	☐	Palaeontology and archaeology
☐	☒	Animals and other organisms
☒	☐	Clinical data
☒	☐	Dual use research of concern
☒	☐	Plants

Methods

n/a	Involved in the study	
☐	☒	ChIP-seq
☒	☐	Flow cytometry
☒	☐	MRI-based neuroimaging

Antibodies

Antibodies used	anti-mCherry (1:1000, Abcam #ab205402, Lot unavailable)<br>anti-GFAP (1:500, Dako #Z0334, Lot unavailable)<br>anti-GAD1/2 (1:100, Abcam #ab183999, Lot unavailable)<br>anti-H3K9me3 (1:100, Abcam #ab8898, Lot unavailable)<br>anti-EZH2 (1:100 Cell Signaling #5246, Lot unavailable)<br>anti-SUZ12 (1:100 Cell Signaling #3737, Lot #8)<br>anti-Nucleolin (1:100 Abcam #ab22758, Lot unavailable)<br>anti-H3K27me3 (1:100 Millipore #07-449, Lot #3170806)<br>Rabbit IgG (1:100, Abcam #ab37415, Lot unavailable)<br>anti-GFP (1:1000 Abcam #ab13970, Lot unavailable)<br>anti-CTIP2 (1:1000, Abcam #ab18465, Lot unavailable)

anti-TBR1 (1:1000, Abcam #ab31940, Lot unavailable)
anti-TBR2 (1:1000, Abcam #ab183991, Lot unavailable)
anti-PAX6 (1:1000, Millipore #AB2237, Lot unavailable)
anti-CUX1 (1:500, Santa Cruz Biotech #sc-13024, Lot unavailable)
anti-SATB2 (1:1000, Abcam #ab51502, Lot unavailable)
anti-NeuroD1 (1:200, Santa Cruz Biotech #sc-1084, Lot unavailable)
anti-NeuroD2 (1:1000, Abcam #ab104430, Lot unavailable)
AlexaFluor-546 anti-Chicken (1:1000, Thermo Scientific #A-11040, Lot #2031903)
AlexaFluor-488 anti-Chicken (1:1000; Thermo Scientific #A-11039, Lot #1812246)
AlexaFluor-546 anti-Rabbit (1:1000, Thermo Scientific #A-11035, Lot #442417)
AlexaFluor-647 anti-Rabbit (1:1000, Thermo Scientific #A-21244, Lot unavailable)
AlexaFluor-647 anti-Rat (1:1000, Thermo Scientific #A-21247, Lot unavailable)
AlexaFluor-647 anti-Mouse (1:1000, Thermo Scientific #A-21235, Lot unavailable)
AlexaFluor-546 anti-Rabbit (1:1000, Thermo Scientific #A-11056, Lot unavailable)
AlexaFluor-647 anti-Rabbit (1:1000, Thermo Scientific #A-21447, Lot unavailable)

Validation

anti-mCherry (1:1000, Abcam #ab205402) - validated for ICC/IF by Takahashi A et al. Nat Commun 13:4039 (2022) doi: 10.1038/s41467-022-31728-z.
anti-GFAP (1:500, Dako #Z0334) - validated by C A Haas Lab (PMID: 35031048) and Del Bondio, A., Longo, F., et al. JCI Insight 2023;8(12):e163576. https://doi.org/10.1172/jci.insight.163576.
anti-GAD1/2 (1:100, Abcam #ab183999) - validated for ICC/IF by Lee S et al. C. Nature 568:93-97 (2019). doi: 10.1038/s41586-019-1053-2. Epub 2019 Mar 27.
anti-H3K9me3 (1:100, Abcam #ab8898) - validated for ChIP by Fukuda Y et al. Methods Mol Biol 2577:161-173 (2023). doi: 10.1007/978-1-0716-2724-2_11.
anti-EZH2 (1:100 Cell Signaling #5246) - validated for ChIP by Gu T et al. J Mol Neurosci. 2023 May;73(4-5):225-236. doi: 10.1007/s12031-023-02114-1.
anti-SUZ12 (1:100 Cell Signaling #3737) - validated for ChIP by Müller M et al. Stem Cell Reports. 2022 May 10;17(5):1070-1080. doi: 10.1016/j.stemcr.2022.03.014
anti-Nucleolin (1:100 Abcam #ab22758) - validated for RIP by Percharde M et al. Cell. 2018 Jul 12;174(2):391-405.e19. doi: 10.1016/j.cell.2018.05.043
anti-H3K27me3 (1:100 Millipore #07-449) - validated for ChIP by C D Chen Lab (PMID: 26302868) and Lochmann TL et al. PLoS One. 2015 Jun 8;10(6):e0129647. doi: 10.1371/journal.pone.0129647
Rabbit IgG (1:100, Abcam #ab37415) - validated for ChIP by J L Rinn Lab (PMID: 20673990) and Kamiya M et al. Nat Commun. 2022 Jan 10;13(1):166. doi: 10.1038/s41467-021-27875-4
anti-GFP (1:1000 Abcam #ab13970) - validated for ICC/IF by Nieuwenhuis B et al. Gene Ther. 2023 Jun;30(6):503-519. doi: 10.1038/s41434-022-00380-z
anti-CTIP2 (1:1000, Abcam #ab18465) - validated for ICC/IF by Morelli KH et al. Nat Neurosci. 2023 Jan;26(1):27-38. doi: 10.1038/s41593-022-01207-1.
anti-TBR1 (1:1000, Abcam #ab31940) - validated for ICC/IF by Wei C et al. Neurosci Bull. 2022 Mar;38(3):249-262. doi: 10.1007/s12264-021-00804-7
anti-TBR2 (1:1000, Abcam #ab183991) - validated for ICC/IF by Lee D et al. Cancer Res Treat. 2023 Jan;55(1):167-178. doi: 10.4143/crt.2022.094
anti-PAX6 (1:1000, Millipore #AB2237) - validated for ICC/IF by Vong KI et al. Mol Brain. 2015 Apr 12;8:25. doi: 10.1186/s13041-015-0115-0
anti-CUX1 (1:500, Santa Cruz Biotech #sc-13024) - validated for ICC/IF by Carabalona A et al. Nat Neurosci. 2016 Feb;19(2):253-62. doi: 10.1038/nn.4213
anti-SATB2 (1:1000, Abcam #ab51502) - validated for ICC/IF by Salamon I et al. Cereb Cortex. 2022 Mar 30;32(7):1494-1507. doi: 10.1093/cercor/bhab302
anti-NeuroD1 (1:200, Santa Cruz Biotech #sc-1084) - validated for ICC/IF by Hong S et al. Cell Death Dis 10, 943 (2019). https://doi.org/10.1038/s41419-019-2174-0
anti-NeuroD2 (1:1000, Abcam #ab104430) - validated for ICC/IF by Tomasello U etal. Cell Rep. 2022 Feb 15;38(7):110381. doi: 10.1016/j.celrep.2022.110381

Animals and other research organisms

Policy information about studies involving animals; ARRIVE guidelines recommended for reporting animal research, and Sex and Gender in Research

Laboratory animals

10-12 week old wild-type C57BL/6J and CD1 IGS mice were purchased from Charles River and delivered to our facility at day 15 (for C57BL/6J) or day 10 (for CD1 IGS) of gestation. They were housed at Istituto Italiano di Tecnologia (IIT) under a 12-hours light-dark cycle at a temperature ranging from 18 to 23°C and 40-60% humidity-controlled environment with ad libitum access to food and water. Pregnant CD1 IGS mice were anesthetized with isofluorane before in utero electroporation and sacrificed by carbon dioxide inhalation followed by decapitation prior to E12, E14 or E18 embryo extraction and histological preparations. Pregant C57BL/6J were sacrificed by carbon dioxide inhalation followed by decapitation prior to E17.5 embryo extraction and preparation of cortical neuron cell cultures.

Wild animals

Our study did not involve wild animals

Reporting on sex

In vivo and in vitro experiments reported in this study were carried out on mouse embryos of both sex since cellular and molecular steps determining the development of the brain cortex do not depend on sex. Also, there is no literature about differences in the expression of L1 elements due to sex. Furthermore, in vitro experiments are performed on cells deriving from a pool of brain-derived

cells isolated from different embryos belonging to the same litter, and determination of sex of embryos is not compatible with timing of cell isolation from the brain. For these reasons, sex of the embryos was not determine or collected.

Field-collected samples: No field collected samples were used in the study

Ethics oversight: All animal procedures were approved by IIT animal use committee and the Italian Ministry of Health (Animal Study Proposal #693/2019-PR.) and conducted in accordance with the Guide for the Care and Use of Laboratory Animals of the European Community Council Directives.

Note that full information on the approval of the study protocol must also be provided in the manuscript.

Plants

Seed stocks: Not applicable

Novel plant genotypes: Not applicable

Authentication: Not applicable

ChIP-seq

Data deposition

☒ Confirm that both raw and final processed data have been deposited in a public database such as GEO.

☒ Confirm that you have deposited or provided access to graph files (e.g. BED files) for the called peaks.

Data access links
May remain private before publication.: PRJEB58556

Files in database submission: ERR10694672, ERR10694673, ERR10694674, ERR10694675, ERR10694676, ERR10694677, ERR10694678, ERR10694679, ERR10694680, ERR10694681, ERR10694682, ERR10694683, ERR10694684, ERR10694685, ERR10694686, ERR10694687

Genome browser session
(e.g. UCSC): no longer applicable

Methodology

Replicates: ChIP-Seq experiment was performed on mouse cortical cells infected with AAV expressing shCtrl or shL1-a. Input, GFP, rabbit control IgG, H3K27me and Ezh2 were evaluated. One technical replicate for each independent biological sample (n=2) was sequenced.

Sequencing depth: 60 millions reads per sample.

Antibodies:
anti-GFP (1:100 Abcam #ab290)
anti-H3K27me3 (1:100 Millipore #07-449)
anti-EZH2 (1:100 Diagenode #C15410039-50)

Peak calling parameters: he H3K27me3 and Ezh2 ChIP-Seq reads corresponding to the shL1 and shCTRL samples were aligned to the mm10 mouse genome using the Bowtie2 aligner. The peak calling was done with epic2, a re-implementation of SICER, on the BEDPE files using matched input samples as control and a FDR threshold of 0.01 when calling the peaks or islands.

Data quality: The unmapped reads, not primary aligned reads, and reads aligned with a MAPQ quality score below 30 were filtered out with samtools. The duplicated reads were marked with Picard, then removed and the reads mapped as proper pairs were sorted into a BAM file. The BAM files were transformed into bigWig with deepTools for visualization purposes in the UCSC genome browser and into BEDPE format with bedtools. The data was normalized by default, based on sequencing depth and the differential analysis was also performed by default, using DESeq2 and a FDR threshold of 0.05.

Software:
Bowtie2 aligner
Picard
DiffBind
